# Mixed Epithelial and Stromal Tumor: A Rare Renal Neoplasm—Case Report with Clinicopathologic Features and Review of the Literature

**DOI:** 10.1155/2023/3528377

**Published:** 2023-01-04

**Authors:** Mukund Tinguria, Katherine Chorneyko

**Affiliations:** Department of Pathology and Laboratory Medicine, Brantford General Hospital, 200 Terrace Hill Street, Brantford, Ontario, Canada Postal Code-N3R 1G9

## Abstract

Mixed epithelial and stromal tumor (MEST) is a rare benign renal neoplasm composed of epithelial and stromal components. Here, we report a 61-year-old woman presenting with a left complex cystic renal mass. The lesion was found incidentally on ultrasound for abdominal discomfort. CT scan and MRI showed a 7.4 cm complex cystic lesion in the left kidney. The differential diagnoses included complex renal cyst and cystic renal cell carcinoma. Laparoscopic nephrectomy showed a large 7.5 cm multicystic tumor with thick and thin septae and smooth walled-cysts containing clear watery fluid. Histologic examination showed variable sized cysts lined by flattened, cuboidal to columnar epithelium with focal hobnailing. No significant cytologic atypia or mitoses were noted. The cyst lining epithelium was positive for CK7 and high molecular weight cytokeratin (34Be12). The stroma was positive for alpha smooth muscle actin, CD10, estrogen receptor, and progesterone receptor. This report contributes an additional case to our collective knowledge of these lesions and summarizes the literature around these rare neoplasms.

## 1. Introduction

Renal mixed epithelial and stromal tumors (MEST) along with adult cystic nephroma (ACN) are rare renal neoplasms that are included in the mixed epithelial and stromal tumor family in the 2022 WHO classification of renal neoplasms. These are mostly benign renal neoplasms with infrequent local recurrences reported. Rarely, there could be malignant transformation of the epithelial or stromal component. Imaging studies show complex cystic lesions, but these cannot be further subclassified accurately as there are no distinguishing features of mixed epithelial and stromal tumors on imaging studies. Histological and immunohistochemical studies are required for a definite diagnosis. We describe a case of MEST in a 61-year-old woman who presented with a left complex cystic renal mass. The pathologic and immunohistologic features of this rare renal neoplasm are described along with a review of literature. Differential diagnoses, theories of histogenesis, and molecular genetic mechanisms are also discussed.

## 2. Materials and Methods

### 2.1. Case Report

A 61-year-old female patient presented with vague abdominal discomfort. A left-sided large complex renal cyst was found incidentally on ultrasound examination leading to subsequent CT scan examination. The left kidney on CT scan measured 11 cm in length. The complex cystic lesion was present at the upper pole measuring 7.3 cm × 6.3 cm There were thin faint septations throughout the lesion with some septations showing enhancement. The lesion had well-defined margins, and it compressed nondilated upper and midpole calyces. There was no evidence of hydronephrosis, renal calculi, or other focal renal lesions. There was no evidence of ureteric obstruction or ureteric calculi. CT impression was that of a complex renal cyst or a cystic renal neoplasm (cystic renal cell carcinoma). Apart from a 6.4 cm right simple renal cyst, there were no other significant intra-abdominal or pelvic findings on CT imaging. Subsequent MRI examination confirmed CT scan findings and, in addition, showed no abnormality in the perinephric fat, left renal vein, or lymph nodes. MRI impression was that of a predominantly cystic renal cell carcinoma. Of note, there was no family history of renal neoplasms in the patient.

Laparoscopic left nephrectomy was carried out. The patient's postoperative course was unremarkable. She has been regularly followed up by annual imaging studies of the thorax, abdomen, and pelvis. There is no evidence of tumor recurrence or metastases after four years of initial diagnosis. The right-sided simple renal cyst is also stable on imaging studies.

### 2.2. Pathology

**Figure 1:**
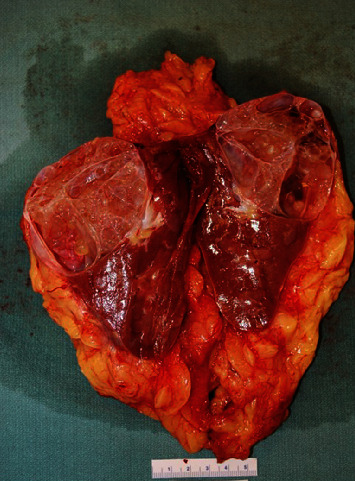
Gross appearance of the tumor. A well-circumscribed solid and cystic neoplasm is present at upper pole of the kidney and shows cysts of variable sizes on cut surface. The cysts were filled with clear watery fluid.

The specimen received for pathological examination consisted of a kidney with perinephric fat altogether weighing 554 grams. The kidney per se measured 12.3 cm superior to inferior, 8.3 cm medial to lateral, and 5.5 cm anterior to posterior. A large multiloculated complex cystic lesion was present in the upper pole (Figure 1), measuring 7.5 cm medial to lateral, 6.3 cm superior to inferior, and 5.4 cm anterior to posterior. The lesion was confined to kidney with no extension into the perinephric adipose tissue. On sectioning, the cut surface demonstrated multiloculated cystic and solid lesion with cysts of variable sizes ranging from 0.3 to 3.0 cm, with thick and thin septae between them. The cysts were filled with clear watery fluid. There were no areas of hemorrhages and necrosis. There was no invasion of the renal pelvis or hilar blood vessels. The uninvolved kidney was grossly unremarkable with well-defined corticomedullary junction. The renal pelvis and calyces were grossly unremarkable. No adrenal gland or lymph nodes were present in the perinephric adipose tissue. The lesion was extensively sampled for histological examination.

**Figure 2:**
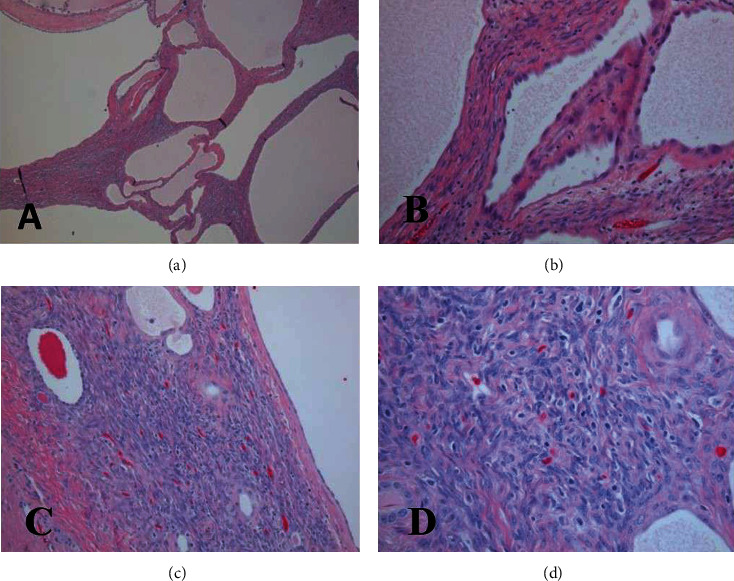
(a) Hematoxylin and eosin sections from the neoplasm. The tumor consists of cysts of variable sizes separated by septae and mesenchymal stroma (40x). (b) Higher magnification from the tumor showing cysts lined by flattened and cuboidal epithelium with hobnail appearance of nuclei in some areas (100x). (c) Ovarian type stroma is present in some areas (40x). (d) Higher magnification showing ovarian type stroma (100x).

Histological examination showed multiple cysts separated by intervening stroma consisting of elongated spindle cells which resembled ovarian type stroma in many areas (Figure 2). The cysts were lined by cuboidal to columnar cells with focal hobnail cells, and in many areas, the lining was markedly attenuated and flattened. The stroma showed thick-walled blood vessels. No cellular atypia was noted. No mitoses were identified. The adjacent kidney, renal pelvis, hilum, and ureter were unremarkable.

**Figure 3:**
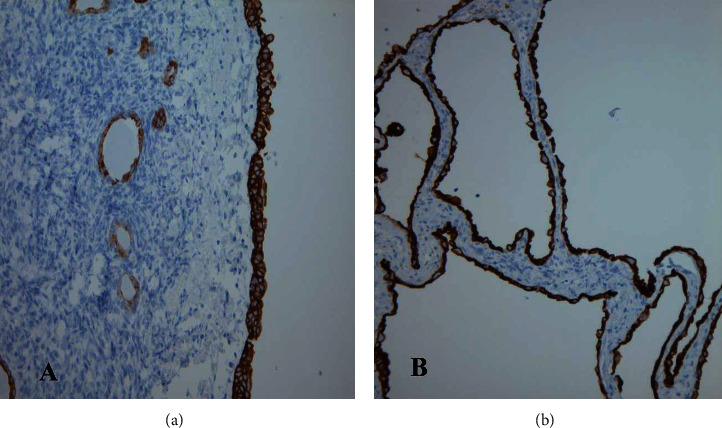
Immunohistochemical staining. (a) The cyst lining epithelium showing positivity for HMWCK (100x). (b) Positivity for CK7 in the cyst lining epithelium (100x).

**Figure 4:**
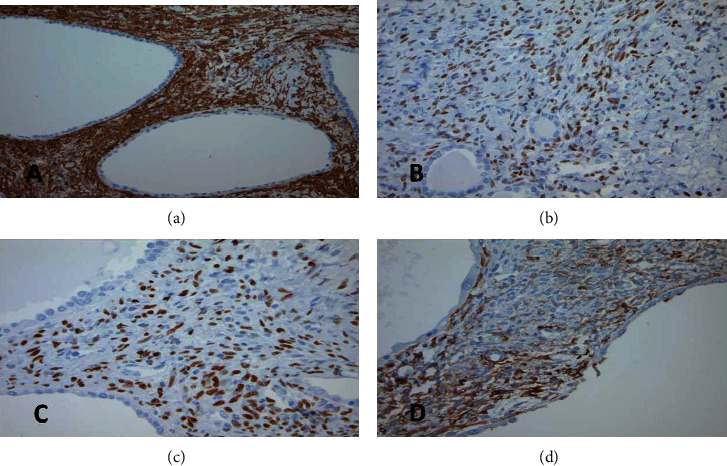
Immunohistochemical staining. The stroma showing positivity for (a) smooth muscle actin (SMA, 200x), (b) estrogen receptor (ER, 200x), (c) progesterone receptor (PR, 200x), and (d) CD10 (CD10, 200x).

Immunohistochemical studies demonstrated positivity for CK7 and high molecular weight cytokeratin (34BE12) of the cyst lining (Figure 3). The stromal cells showed positivity for ER (estrogen receptor), PR (progesterone receptor), CD10, and SMA (smooth muscle actin) (Figure 4). The immunohistochemical profile was in keeping with the diagnosis of mixed epithelial and stromal tumor (MEST).

## 3. Discussion

Mixed epithelial and stromal tumor of the kidney (MEST) is an uncommon neoplasm composed of variable cystic and solid components with diverse epithelial and stromal elements. It was first described by Michal and Syrucek in 1998 as a distinctive tumor composed of well-differentiated ducts with a surrounding spindle cell stroma [1] and has subsequently been referred to by various names such as adult mesoblastic nephroma, cystic hamartoma of renal pelvis, leiomyomatous renal hamartoma, and renal epithelial and stromal tumor (REST). It typically occurs in perimenopausal women often with long-term hormonal replacement therapy. The female to male ratio is 7 : 1 [2]. The age range is from 36 to 80 years (mean 49.7 years) [3]. The tumor in most cases is unilateral, although Sangoi and Higgins [4] reported the first case of bilateral MEST in a 41-year-old dialysis dependent woman with end stage renal disease. Suzuki et al. [5] reported the first case of malignant MEST in a male patient. An association with estrogen exposure has been suggested because of their occurrence in women on hormonal replacement therapy and in men receiving androgen deprivation or estrogen therapy for prostate cancer. There was no history of hormone replacement therapy in our patient.

Clinically and radiologically, it mimics other cystic renal neoplasms, especially cystic renal cell carcinoma, necessitating histopathological examination and immunohistochemical studies for definite diagnosis [6]. The tumor is often incidentally found, although in some instances, patients present with abdominal/flank pain, hematuria, or urinary tract infection [3].

The clinicopathologic features of MEST described in the literature are summarized in Table 1. The tumors range from 0.3 cm to 21 cm [3, 7–12]. Grossly, these tumors are generally solitary, unencapsulated but well circumscribed with a mixture of solid and cystic components. The cystic areas consist of noncommunicating clear serous fluid-filled cysts of variable sizes and shapes with smooth glistening linings. Adult cystic nephroma (ACN) is a predominantly cystic tumor with very little or no solid component and generally felt to be within the spectrum of the MEST family of tumors. Antic et al. [3] in their study of 15 cases (8 MEST and 7 cystic nephromas) found that most cases involved renal cortex with only 2 cases of MEST involving renal pelvis.

Histologically, MEST is composed of a stromal and an epithelial component. The stroma is paucicellular to hypercellular and shows diverse morphologies ranging from dense collagenous to edematous and fibrous stroma composed of slender to plump stromal cells. Areas of smooth muscle differentiation or ovarian type of stroma can be seen as well as stromal condensation around epithelial components. The stroma may also show slit-like to thick-walled blood vessels, and on occasion, foamy histiocytes and calcifications may be present. In the largest study of 53 MEST tumors [7], the investigators found a high degree of stromal variability with more than 50% tumors containing more than four different types of stromal elements. This included hypocellular and cellular fibrous stroma, smooth muscle, edematous stroma, hypervascular stroma, and stroma with sclerotic blood vessels. Adipose tissue was present in 34%. A hypocellular stroma predominated in patients with larger tumors, whereas cellular stroma predominated with smaller tumors.

The epithelial component of MEST consists of cysts and glands of varying sizes. The gland and cyst lining epithelium also displays a broad spectrum of morphology ranging from flat, cuboidal, columnar, epithelium to hobnail, urothelial-like, clear cells, and ciliated epithelium. Combinations of different epithelial elements are commonly seen. The number of epithelial elements found is directly proportional to the number of blocks analyzed, indicating the importance of generous sampling [7]. Cytologic atypia is absent to minimal. Mitoses, hemorrhages, and necrosis are infrequent; however, it should be noted that rare cases of malignant transformation have been reported. Jung et al. [13] described 2 cases of MEST of the kidney with malignant transformation. Sukov et al. [14] described a case of malignant mixed epithelial and stromal tumor of the kidney with rhabdoid features. In addition, MEST can coexist with renal cell carcinoma. Mudaliar et al. [15] described a biphasic renal tumor consisting of MEST with focal papillary renal cell carcinoma, while Arriola et al. [16] described a case of malignant MEST with 2 simultaneous renal cell carcinomas in a male patient.

The immunohistochemical features of MEST reported in the literature are summarized in Table 1. The epithelial component shows positivity for cytokeratins, GATA3, PAX2, and PAX8. In most tumors, the stroma is positive for smooth muscle actin (SMA), desmin, caldesmon, ER and PR, and CD10. Inhibin, calretinin, FOXL2, HMB45, Melan A, WT1, CD34, and S100 are noted in the stroma in some studies. Mai et al. [9] reported positivity for HMB 45 in the stromal component in one of the fourteen cases studied, and they suggested that this case might represent a PEComatous variant of MEST.

It is important to differentiate MEST from cystic renal cell carcinoma, Wilms' tumor, monophasic spindle cell tumors like leiomyoma, solitary fibrous tumor, and metanephric adenofibroma. Angiomyolipoma with epithelial cysts (AMLEC) and rare cases of renal endometriosis should also be considered in the differential diagnosis of MEST. Unlike renal cell carcinoma, epithelial component of MEST rarely forms sheets, and spindle cell areas of MEST may be cellular but lack pleomorphism, cytologic atypia, necrosis, and mitotic figures that are typical of sarcomatoid renal cell carcinoma. MEST can be distinguished from Wilms' tumor, and cystic partially differentiated nephroblastoma by the lack of blastema and other distinctive morphologic and clinical features of MEST. Monophasic spindle cell tumors such as leiomyoma and solitary fibrous tumor rarely occur in the kidney and should be ruled out by careful examination and thorough sampling of the tumors. The smooth muscle component of angiomyolipoma is like that of MEST; however, glandular elements are usually not noted in angiomyolipoma and when rarely identified, represent entrapped tubules. Metanephric adenofibroma, a rare biphasic renal lesion, can be differentiated from MEST based on clinical and pathologic features. Metanephric adenofibroma usually occurs in children and young adults. The epithelial components of metanephric adenofibroma do not usually show cystic component, and when seen is focal. The spindle component is usually negative for both actin and desmin in adenofibroma.

Renal angiomyolipomas are mesenchymal tumors that often present in the kidneys on a background of underlying tuberous sclerosis, a rare autosomal dominant disease [17]. As angiomyolipomas are generally solid tumors, they do not enter the differential diagnoses of adult cystic renal neoplasms. However, AMLEC, a distinctive variant of angiomyolipoma bearing grossly apparent epithelial cysts and a cellular mullerian-appearing subepithelial stroma, has been reported. [12, 18–23]. There are many similarities between MEST and AMLEC. Both the tumors are characterized by cystic and solid component in variable distribution. Smooth muscle fascicles and cysts lined by hobnail or columnar epithelium are common features of MEST and AMLEC. Additionally, stromal cells of MEST and AMLEC are immunoreactive for smooth muscle actin, desmin, ER, and PR. There are, however, several differences between MEST and AMLEC. MEST occurs predominantly in women and male patients with long-term history of estrogen exposure. In contrast, AMLEC occurs in men without history of exogenous hormone exposure. The vessels of AMLEC have dysplastic features which are not seen in MEST. Smooth muscle cells in the wall of AMLEC form less well-developed fascicles, are often epithelioid, and have clear cytoplasm than those of MEST. Most importantly, MEST is negative for melanocytic markers HMB45 and Melan A.

Urinary tract endometriosis is exceedingly rare. Most cases of urinary tract endometriosis involve bladder followed by ureter, renal involvement being extremely rare. [24–30]. Renal endometriosis may present as flank pain and hematuria like renal neoplasms. There may be a significant mass effect which can mimic malignancy on imaging studies. Microscopic features of renal endometriosis are similar to those seen in other body sites with inactive or proliferative endometrial glands surrounded by endometrial stroma. The stroma in endometriosis is different than the diverse stromal component seen in MEST. The endometriosis may be associated with areas of cystic change and foamy and hemosiderin laden macrophages indicating old hemorrhages. Sometimes, there may be prominence of smooth muscle or a fibrotic reaction. The immunohistochemistry demonstrates positivity for CK7, ER, PR, PAX8, CA125, and vimentin in the glandular epithelial cells. The stroma shows positivity for vimentin, ER, PR, and CD10.

Molecular studies showing similar mRNA expression profile between adult cystic nephroma (ACN) and mixed epithelial and stromal tumor (MEST) indicate that these tumors represent opposite ends of the same disease spectrum [31]. Jin-Ping et al. [32] described a mixed epithelial and stromal tumor in a 75-year-old man and two of his daughters and compared histological and molecular profile in 2 familial and 3 sporadic cases. They concluded that MEST could occur in a familial and hereditary setting. There was no family history of MEST in our patient. The histological and molecular profile of the hereditary and sporadic cases was similar in their studies. Vanecek et al. [33] conducted mutation analysis of DICER 1 gene in 29 cases of mixed epithelial and stromal tumor/adult cystic nephroma, which included 1 case of malignant mixed epithelial and stromal tumor/adult cystic nephroma with sarcomatoid differentiation of the stromal component. All 29 cases studied using polymerase chain reaction and direct gene sequencing were negative for mutations in DICER 1 hot spot codon 1705, 1709, 1809, 1810, 1813, and 1814. Their results concluded that mixed epithelial and stromal tumor/adult cystic nephroma has no relation to pediatric cystic nephroma on a molecular genetic level. A sizable portion of pediatric cystic nephroma carries DICER 1 gene mutations in the form of either loss of function or missense mutations [34, 35].

Calio et al. [7] conducted clinicopathologic study of 53 cases of MEST and considered MEST and adult cystic nephroma as separate entities. Areas like adult cystic nephroma were present in 6 out of the 53 cases they studied. Vast majority (74%) of their cases showed cystic component, and only 26% of the cases were composed exclusively of solid component. In the 2022 WHO classification of renal neoplasms [36], MEST is classified as MEST family along with adult cystic nephroma, and this is supported by molecular studies.

The definite histogenesis of mixed epithelial and stromal tumor/adult cystic nephroma is not known. Based on ovarian type stroma and Mullerian-related immunohistochemical profile, Turbiner et al. [8] suggested that these tumors may originate from Mullerian remnants misplaced during embryogenesis. Tickoo et al. [37] observed estrogen and progesterone receptor positive stroma in some nonneoplastic kidneys as well as tumor-bearing kidneys away from the tumor. The only common findings in all cases were generalized or segmental hydronephrosis or tumor compression-related focal obstruction. This suggested that it may represent a metaplastic change in the renal interstitial cells surrounding obstructed epithelial structures. Based on Mullerian-like stroma and epithelial component displaying features of Mullerian epithelium, Mai et al. [9] suggested that the mixed epithelial and stromal tumor represented a tumor developing from Mullerian-like stromal cells, and this stroma had the potential of stimulating growth of renal tubules by contact with development into cysts, and Mullerian stroma likely induces the renal tubules to differentiate into Mullerian-like epithelium. Adsay et al. [38] postulated that a deranged hormonal milieu (perimenopausal changes or therapeutic hormones with unopposed estrogen) induces proliferation of mesenchyme, which in turn drives the growth of the epithelial component. They also propose that possible cell of origin for these tumors is the primary mesenchyme which has the capacity for dual (mesenchymal and epithelial) differentiation.

Extensive cystic renal neoplasms cannot be distinguished on preoperative imaging studies. A preoperative biopsy and intraoperative frozen section analysis do not lead to a correct diagnosis in many cases. Fortunately, the operative strategy is the same for all these tumors. In such cases, nephron sparing surgery is indicated whenever technically feasible as almost all extremely cystic neoplasms have a good prognosis.

## 4. Conclusion

Mixed epithelial and stromal tumor (MEST) is a rare benign renal neoplasm composed of epithelial and stromal component and shows variable solid and cystic areas. Along with adult cystic nephroma, it is included in the family of mixed epithelial and stromal tumor in the 2022 WHO classification of renal neoplasms. Most tumors are benign; however, they may recur if incompletely excised. Rare examples of malignant transformation of the epithelial or stromal component have been reported. This case emphasizes the value of histological examination; as radiologically, there are no features to distinguish it from other cystic renal lesions including cystic renal cell carcinoma.

## Figures and Tables

**Table 1:** Summary of reported cases of MEST.

Authors/date	Number of cases	M : F	Age (years)	Tumor size (cm)	Histologic findings	Immunohistochemical findings
Michal M et al., 1998	1	F	48	10	Well-differentiated ducts with surrounding spindle stroma	Epithelium–Cytokeratins Stroma–HHF35, smooth muscle actin, and desmin

Adsay N et al., 2000	12	1 : 11	31–71 (mean 56)	3–12 (mean 6 cm)	Epithelium: glands–widely scattered or crowded, dilated, and showing complex branching architecture and papillae. Lined by cuboidal, flattened or hobnail cells, rare ciliated epithelium. Stroma: wide range- paucicellular fibrous to hypercellular with fascicular arrangement. Keloid-like resembling solitary fibrous tumor. Condensation of stroma around cystic epithelial component. Some cases showed prominent vasculature with thick-walled and dilated blood vessels. Fat cells (1 case)	Epithelium: cytokeratin (AE1/AE3) CAM 5.2 vimentin (4 cases) Stroma: vimentin SMA Desmin ER PR (3 cases; all female) Both epithelial and stromal component negative for S100, HMB 45, and CD34.

Michal M et al., 2004	24	1 : 11	41–75 (mean 54)	1–14 (mean 6.7)	Epithelium: cysts lined by flat to hobnail type cells. Other less frequent epithelial components include endometrioid, tubal, clear cells, and squamous type. Stroma: Spindle cells resembling ovarian stroma	Epithelium: cytokeratins variable ER and PR positivity Stroma: Vimentin. SMA, desmin (patchy), ER, PR Glands and stroma: negative for S100, chromogranin, neurofilament, synaptophysin, inhibin, and HMB45

Antic T et al., 2006	15	01 : 02.7	22-80 (mean MEST 49.7,Mean ACN 50.4	0.5-10 (mean MEST 2.9, ACN 4.3)	Epithelium: Cysts and tubules lined by flattened, cuboidal, columnar epithelium. Focal hobnail appearance. Stroma: Spindle cells With variable cellularity, myxoid, myofibroblastic, fibrous, adipocytes, and calcifications	Epithelium: positive for pan-cytokeratin (AE1/AE3), CK7, HMWCK (variable), CD10 (variable), negative for ER and PR stroma: Positive for SMA, WT1 (variable), CD34 (variable), ER (variable), PR (variable), negative for CD117, HMB 45, inhibin

Jevremovic et al., 2006	11	01 : 04.5	43–69 (mean 57)	3.2–12 (mean 6.8)	Epithelium: multilocular cysts lined by single layered epithelium. Hobnail epithelium. No epithelial atypia. Stroma: Spindle cells, ovarian-like stroma No stromal atypia.	Epithelium: pn-cytokeratin (AE1/AE3) cam 5.2 Stroma: vimentin,SMA,desmin (focal), ER, PR

Mai K et al. 2007	14	1 : 06	39–70 (mean 55+/-9)	2.5–12 (mean 5.2+/-2.9)	Epithelium: Cysts, microcysts lined by flat, columnar to hobnail cells. Rarely ciliated and urothelial-like lining cells. Stroma: Spindle cells with smooth muscle tissue, ovarian type stroma, and fibrous tissue.	Epithelium: pan-cytokeratin (AE1/AE3), CK7 ER–variable PR–variable CD10–variable Stroma: Vimentin, SMA, ER, PR, CD10, HMB45 (2 cases) (one case showing collision tumor with AML and HMB-45 positivity noted in the AML component, and second case considered PEComatous variant of MEST)

Turbiner J et al., 2007	34	1 : 16	24–84 (mean MEST 52, ACN 48)	2 to 21 (mean MEST 9.7, ACN 7.3)	Epithelium: Variable sized cysts and phyllodes like glands, lined by flat to cuboidal To hobnail cells stroma: hypocellular, hypercellular, collagenized, ovarian type, periglandular hyalinization, prominent vascularity, smooth muscle metaplasia, calcification, and hemorrhage.	Epithelium: focal positivity for CD10, ER negative for inhibin, calretinin Stroma: R, PR, CD10, inhibin (focal), calretinin

Sukov W et al., 2007	1	F	84	10.5	Showed features of malignant mixed epithelial and stromal tumor with rhabdoid features epithelium: multiple tubules and variably sized cystic structures lined by benign epithelium. Stroma: malignant appearing spindle stroma resembling ovarian stroma and stroma showing extensive rhabdoid differentiation	Epithelium: pan-cytokeratin (AE1/AE3), CK7, Cam5.2, EMA Stroma: WT1, CD99, CD56, IN1, ER, SMA (focal)

Jung et al., 2008	2	F	53 and 56	6 and 13	Case#1 Epithelium: Cysts of variable sizes lined by single layer of cuboidal cells and hobnail cells.No cytologic atypia. Tubules lined by bland cells Stroma: Spindle cells ovarian-like stroma In addition, showed malignant stroma with increased cellularity, atypia, and frequent mitoses. Case #2 Epithelial component-like case #1 Focal carcinomatous component with undifferentiated large cells Stroma: In addition to benign and malignant appearing stroma-like, case #1 also showed areas resembling fibrosarcoma, undifferentiated sarcoma, cellular myxoid sarcoma, chondrosarcoma, and rhabdomyosarcoma	Case #1 epithelium: pan-cytokeratin (AE1/AE3) Stroma: Vimentin, PR, negative for SMA, S100, ER Sarcomatous areas negative for ER and PR Case #2 epithelium: cytokeratin AE1/AE3 EMA Stroma: vimentin, PR (focal), negative for ER Rhabdomyosarcomatous areas: SMA, desmin Undifferentiated sarcomatous areas: CD99, Bcl-2, CD10, S100 Chondrosarcomatous areas: S100

Sangoi A et al., 2008	1	F	41	Bilateral tumors Right–5 Left-7.8	Right and left-sided tumors showed identical features epithelium: Glands, cysts lined by flattened to hobnail to urothelial-like cells, papillae, and phyllodes like areas. Stroma: Edematous, cellular, ovarian-like, hyalinized, smooth muscle like morphology, myxoid, calcification, and adipose tissue	Epithelium: pan-cytokeratin (AE1/AE3) cam 5.2 Stroma: ER, PR, desmin, negative for HMB45

Suzuki T et al., 2013	1	M	67	3.5	Epithelium: Cystic spaces lined by cuboidal epithelium with occasional ciliated cell stroma: Malignant stroma with atypical small round cells with a high nuclear cytoplasm ratio and spindle cells without cytologic atypia (malignant-mixed epithelial and stromal tumor of the kidney	Epithelium: ctokeratin vimentin ER PR Stroma: vimentin, Bcl-2, CD99, CD10 (<10% of cells), negative for SMA and S100

Mudaliar et al., 2014	1	F	75	8.1	(MEST with focal papillary renal cell carcinoma) Epithelium: Cysts lined by flattened to cuboidal to hobnail cells One cystic cavity lined by papillary proliferation lined with dysplastic cuboidal epithelium with variable cytologic atypia ranging from mild to frank dysplasia and necrosis. Stroma: Spindle cell, ovarian-like stroma. No stromal atypia, mitoses, or necrosis	Epithelium: CK7 CD10 (focal) papillary areas CK7 PR (focal) Stroma: vimentin, SMA, ER, PR, CD10 (focal), negative for inhibin and HMB45

Sun B et al., 2015	1	F	32	3.8	Epithelium: Variably sized cysts lined by cuboidal epithelium with focal hobnailing, without significant cytologic atypia and mitosis. Stroma: Ovarian-like stroma was condensed around the cysts and was variably cellular with areas of muscle differentiation and thick-walled vessels	Epithelium: CK19, HMWCK and alpha methyl COA racemase Stroma: SMA, desmin in areas of muscle differentiation, ER, PR, CD10, negative for HMB45

Calio A, et al., 2016	53	1 : 6.6	18–82 (median age 51)	0.3-17.5 (median 4.0)	Epithelium: Crowded glands, branching ducts, small glands with tall cuboidal cells, thyroid follicle-like glands, spatulate and complex papillae, Stroma: Hypocellular fibrous, cellular, smooth muscle, edematous, hypervascular, sclerotic walled vessels, and adipose tissue	Epithelium: ER (10%) PR (10%) CD10 (10%) PAX8 (100%), and GATA3 (57%) Stroma: SMA (97%), desmin (89%), caldesmon (88%), ER (88%), PR (95%), CD34 (47%), WT1 (26%), CD10 (90%), S100, HMB45, Melan A, PAX8, GATA3, and inhibin, SF1 (0%)

Arriola A 2018	1	M	61	11.5	Malignant MEST with 2 simultaneous renal carcinomas: Conventional papillary renal cell carcinoma (PRCC) and clear cell renal cell carcinoma (CCRCC) Epithelium: Variable sized cysts lined by flat, cuboidal to hobnailed and bland epithelial cells. Areas of conventional papillary carcinoma (size = 2.0 cm) and clear cell renal cell carcinoma (size = 1.0 cm) Stroma: Loose and cellular ovarian-like stroma. Stroma in some areas showed marked cytologic atypia and increased mitoses (up to 8 mitoses/10 high power field) and necrosis.	Epithelium: epithelium lining cystic spaces: pan-cytokeratin CK7 PAX 8 (focal) negative for CAIX PRCC: CK7 racemase negative for CAIX CCRCC: CAIX negative for CK7 and racemase stroma: vimentin SMA, negative for ER, PR, MHB45, STAT6, TLE, myogenin, and desmin

Tinguria M. 2023 (current study)	1	F	61	7.3	Epithelium: Cysts of variable sizes lined by cuboidal, columnar, and flattened epithelium with focal hobnailing. Stroma: cellular spindle cells resembling ovarian stroma. No cytologic atypia. No mitoses.	Epithelium: CK7, HMWCK, Stroma: ER, PR, CD10, SMA

Note: Cases classified as adult cystic nephroma and MEST separately in some studies have been grouped together as MEST in this table following 2022 WHO classification. ER: estrogen receptor, PR: progesterone receptor, SMA: smooth muscle actin, AML: angiomyolipoma, PEC: perivascular epithelioid cells, PRCC: papillary renal cell carcinoma, CCRCC: clear cell renal cell carcinoma, CAIX: carbonic anhydrase IX, STAT 6: signal transducer and activation of transcription (6), and TLE: transducin-like enhancer of split.

## Data Availability

The data used to support the findings of this study are included within the article.
